# Detection of Intratumor Heterogeneity in Modern Pathology: A Multisite Tumor Sampling Perspective

**DOI:** 10.3389/fmed.2017.00025

**Published:** 2017-03-06

**Authors:** Jesús M. Cortés, Giovanni de Petris, José I. López

**Affiliations:** ^1^Quantitative Biomedicine Unit, Biocruces Research Institute, Barakaldo, Spain; ^2^Ikerbasque: The Basque Foundation for Science, Bilbao, Spain; ^3^Department of Cell Biology and Histology, University of the Basque Country (UPV/EHU), Leioa, Spain; ^4^Department of Pathology and Laboratory Medicine, Penrose St Francis Hospital, Colorado Springs, CO, USA; ^5^Department of Pathology, Cruces University Hospital, Barakaldo, Spain; ^6^Biomarkers in Cancer Unit, Biocruces Research Institute, Barakaldo, Spain; ^7^University of the Basque Country (UPV/EHU), Leioa, Spain

**Keywords:** tumor sampling, intratumor heterogeneity, stomach, large bowel, urinary bladder, *in silico* modeling

## Abstract

Current sampling protocols of neoplasms along the digestive tract and in the urinary bladder have to be updated, as they do not respond to the necessities of modern personalized medicine. We show here that an adapted version of multisite tumor sampling (MSTS) is a sustainable model to overcome current deficiencies in digestive and bladder tumors when they are large enough so as to make unaffordable a total sampling. The new method is based on the divide-and-conquer algorithm and includes a slight modification of the MSTS, which proved to be useful very recently in clear cell renal cell carcinoma. This *in silico* analysis confirms the usefulness of MSTS for detecting intratumor heterogeneity (ITH) in tumors arising in hollow viscera. However, MSTS does not seem to improve routine traditional sampling in detecting tumor budding, extramural venous invasion, and perineural invasion. We conclude that (1) MSTS is the best method for tumor sampling to detect ITH balancing high performance and sustainable cost, (2) MSTS must be adapted to tumor shape and tumor location for an optimal performance.

## Introduction

Neoplasia has been proposed as a model of intracellular metabolic shift (1), where cells adopt an anaerobic glycolysis-dependent metabolism to sustain their uncontrolled proliferation, a phenomenon known as the Warburg effect (2). Molecular advances in the last years have revealed the extreme complexity of human tumors (3), and that intratumor heterogeneity (ITH) is a reflection of this complexity. ITH is obtaining an extraordinary importance in modern oncology during the last years (4, 5). Characteristically, ITH follows a stochastic spatial distribution along different tumor regions (6) making every single case really unique and unrepeatable. However, ITH is not a time-related tumor acquisition. In fact, a recent work has demonstrated in renal cancer that ITH appeared at very early stages in tumor development and therefore was not the result of tumor progression (7). Because ITH is high for most of the malignant tumors, it represents right now a major obstacle for the success of modern targeted therapies (8).

Many big tumors are in practice far away for being totally analyzed. In these situations, pathologists (the medical specialists handling surgical specimens and performing tumor samplings) partly sample such big tumors under the assumption that the selected samples are good representatives of the whole neoplasm. In other words, pathologists decide which parts of the tumors will be selected for histological and molecular analysis and which parts will not (the latter ones usually correspond with the tumor majority). For such a purpose, classic handling and routine sampling protocols (RP) are available for different tumor topographies in referential textbooks (9). However, these protocols were conceived in a dogmatic fashion during a period in which ITH was not a concerning issue and, surprisingly, none of them have been adapted to current necessities so far.

Recent literature shows many examples of controversial results (sometimes contradictory) in molecular analyses of the same tumor types and, even more important, deep disagreements in the performance of some expensive therapies. In this specific scenario, basic researchers, oncologists, and pathologists should first wonder how many of these apparent inconsistencies might be simply due to incomplete tumor samplings.

Representativeness of the tumor sample is an important issue in modern oncology, particularly in those cases in which therapeutic decisions are made with small core biopsies. In these cases, liquid biopsy appears in the next horizon as a promising alternative in detecting tumor heterogeneity and in identifying mutations associated with tumor aggressiveness. Furthermore, liquid biopsy could be regarded as a complementary sample to surgical specimens for the application of massive sequencing tools.

## The Context

An efficient sampling of a large tumor must ensure properly ITH detection without incurring extra costs, and such a method has not yet been established in modern pathology. We have recently suggested a novel method called multisite tumor sampling (MSTS) (10) that makes use of the same number of cassettes and obtain a much better performance in detecting ITH as compared to RP without extra costs. Importantly, MSTS was clinically validated for being more efficient in detecting ITH as compared to RP on a series of clear cell renal cell carcinomas (CCRCCs) (11).

Multisite tumor sampling is based on the divide-and-conquer (DAC) algorithm (12). DAC is a useful strategy to solve complex problems in basic science (13) and has been also applied to answer biological problems (14, 15). In short, the DAC strategy consists of recursively breaking down a problem into smaller parts (divide) until these are simple enough to be solved directly (conquer). Then, partial solutions are merged to solve the original problem.

Multisite tumor sampling was shown to be an efficient method to sample large tumors for neoplasms growing in solid organs, i.e., kidney (10). However, the same protocol can be applied to any other large tumors, like those arising in soft tissues, liver, lung, testis, thyroid, breast, and others.

Some carcinomas, however, do grow with different shapes in the mucosa (excavated, plaque-like, plateau-like, polypoid), not like simple spheroids, and their growth is different depending on the axis considered, i.e., vertical or horizontal. This tumor category is represented, for instance, by carcinomas along the digestive tract and in the urinary bladder.

## The Problem

Gastric adenocarcinomas (GACs), colorectal adenocarcinomas (CRCs), and urothelial cell carcinomas (UCC) of the urinary bladder are substantially different neoplasms developing in similar topographic sites, since the digestive tract and the urinary bladder are hollow viscera with similar walls that include mucosa, submucosa, muscularis propria, and serosa. A significant subset of UCC and many GACs and CRCs are plaque-like shaped neoplasms having two main components of growth: superficial (radial spread) and vertical (deep invasion). Due to the specific characteristics of the local environment, it is likely that a substantial part of the relevant information for these neoplasms such as biological aggressiveness and prognosis are located in the invasion front (16–18). However, current RP for tumor sampling are not emphasizing enough the necessity of analyzing thoroughly this specific zone (9).

### Urinary Bladder

Urothelial cell carcinomas of the urinary bladder are common neoplasms in Western countries. Over new 76,000 cases will occur in USA in 2016 (19). A relatively small number of UCC undergo radical cystectomy after TUR (20). As acknowledged by Chandra et al. (21), many of the recommendations performed in the last consensuses of bladder cancer were not supported by a strong evidence base. ITH has not yet been thoroughly analyzed in high-grade UCC, and the tumor invasion front into the bladder wall, or beyond, has not received much attention for preferential sampling by pathologists (22, 23). However, as it has been reported recently for renal cancer (7) and other neoplasms (24), the tumor periphery and the tumor invasion front may be important sites with distinct microenvironment and/or somatic mutations (17). Although a recent whole exome sequencing study with three metastatic UCCs reports low spatial genomic ITH in the primary tumors (25), these results need to be confirmed in larger series. Variations in grade and methylation status are, however, common findings in UCC, and necrosis, growth pattern (papillary vs flat), and histological subtyping are well-known classic features with impact on survival that deserve proper identification (26, 27).

### Stomach

Although declining in Western countries during the last decades, new 26,000 cases of GACs are expected in USA in 2016 (19). The major responsible for the decrease of incidence are the successful treatment of *Helicobacter pylori* and changes in lifestyle (28). GAC is another paradigmatic example of highly heterogeneous neoplasm (29, 30). Most GACs are sporadic, but a minority of cases are familiar and hereditary taking part of several different clinical settings (31, 32). Lauren classification of GAC (intestinal, diffuse, and indeterminate types) is still in vogue among most pathologists worldwide (33). Recommendations for pathology reporting GAC include staging data and histological predictors of aggressiveness (34). Tumor budding, a change related to local epithelial-to-mesenchymal transition (35), is related with clinical aggressiveness, similar to what happens for most carcinomas arising along the entire digestive tract and head and neck (18, 36).

### Large Bowel

Colorectal adenocarcinoma is one of the most common human cancers in Western countries. Over new 95,000 cases will occur in USA in 2016 (19). High-quality pathology reporting of CRC includes staging data and histological predictors of aggressiveness (37). It is well known the importance of histological tumor grade/differentiation, of invasion depth, and of the tumor distance from the radial margin in the rectum. Less appreciated, but equally important events are tumor budding (18), extramural venous invasion (38), and perineural invasion (39). These changes occur at the tumor invasive front. Molecular tumor characteristics (affected by genetics, epigenetics, stroma, local immune response, vascularization, and hypoxia characteristics) are also used to define prognostic categories (40). Finally, CRC was proven to be a molecularly complex and highly heterogeneous disease at inter- and intratumor levels (41).

## A MSTS Solution

Pathologists around the world every day face the same dilemma when making the sampling of large tumors: where to sample and when to stop sampling. Many tumors that are totally homogeneous by the naked eye might present in fact high levels of molecular ITH, what increases the pressure to pathologists when performing the sampling. Although there exist some classic rules in relation with tumor/non-tumor interface, necrosis, hemorrhage, tumor edges, etcetera, however, focusing on the tumor itself, pathologists are sampling tumors in a quasi-blind fashion following dogmatic rules or local customs and habits, and this situation is no longer acceptable.

The solution for ITH detection must be affordable and workable at the same time, thereby balancing scientific accuracy and cost. A total tumor sampling, when possible, is the ideal solution. However, many tumors are too large and cannot be totally sampled without collapsing the laboratory workflow. In these cases, pathologists must decide how large the sampling must be. Although (as far as we know) there is no official answer to this question at this time, we have recently proposed a method to increase the number of samples in CCRCC while keeping the same cost fixed (42). We showed [*in silico* analysis (10), method implementation (43), and clinical validation (11)] that a better alternative for tumor sampling is possible: MSTS.

Here, we have adapted our method to the topographic peculiarities of stomach, large bowel, and urinary bladder, respectively, and have performed a similar *in silico* approach to the problem with colon cancer as an example. On the one hand, the modeling takes into account both microsatellite stable and unstable tumor categories in CRC. On the other hand, different densities of tumor budding, extramural venous invasion, and perineural invasion at the tumor front of invasion have also been considered in the analysis.

**Figure 1:**
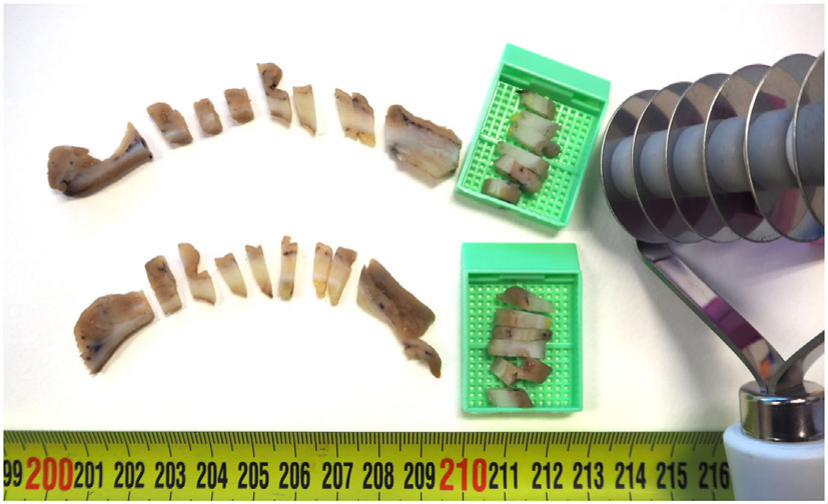
**Practical implementation of multisite tumor sampling in a gastric adenocarcinoma (GAC)**. Two slices of a GAC after the application of multi-wheel rolling pasta cutter obtaining multiple tumor bars that include the full thickness of the tumor (including the front of tumor invasion) that fit six of them in the same cassette (the patient gave written informed consent for the use of this biological material for scientific purposes).

The goal of MSTS is the recruitment of many tumor samples for analysis, but the procedure must be adapted to specific tumor shape. Excavated and plaque-like neoplasms can be sampled with full-thickness bars of tumor tissue including the lower invasion front. Next, six to eight of these bars are placed in the same cassette for study (Figure 1). A thorough analysis of the invasion front into the wall seems mandatory in these tumors, not only for assessing ITH (histological or molecular) at any level in the vertical axis but also to discover tumor budding, and intravascular/perineural invasions, or any other morphologic feature with prognostic implications. Thick plateau-like and polypoid tumors can be sampled with a mixture of bars at the tumor invasion front and cubes at the rest, in a similar way we have previously proposed in CCRCC (10, 11). This way, MSTS is adapted to any possible situation, and many more tumor regions can be sampled without increasing laboratory costs (pathology laboratory costs are calculated by the number of paraffin blocks used for the analysis, where the higher the number of blocks, the higher the cost).

Since obtaining a large amount of these full-thickness tissue bars along several tumor slices may be perceived as too laborious by pathologists, we propose here the use of a multi-wheel rolling pasta cutter (or any similar device) (Figure 1) to get several bars in one step. Similarly, we proposed very recently a potato cutter grid for MSTS implementation in CCRCC (43).

Our accumulated experience with MSTS points to this method as the most affordable sampling strategy in large tumors to guarantee a proper ITH detection without increasing cost. It seems, however, that MSTS must be adapted to the tumor shape, on the one hand, and to the tumor topography, on the other hand. Tumors growing as 3D spheroids like those arising in the kidney, liver, lung, breast, and soft tissues, for example, are not confined by anatomical barriers and are well sampled with tissue cubes, as recently shown (11). However, tumors with more or less flattened shapes, arising in hollow viscera, are confined by well-defined anatomical barriers, namely, muscularis propria. In this context, MSTS must take into account the tumor orientation for sampling to get information at different levels of the wall. Obtaining tumor bars including (if possible) the whole tumor thickness is the best option for these cases.

## *In Silico* Modeling in Favor of MSTS for Hollow Viscera Tumors

We extended here the modeling approach we performed in our previous paper (10), but now the tumor shape is assumed to be a rectangle of dimensions 3 × *L*. So, the ITH modeling is similar to the previous one (10) and is represented by the matrix (γ)*_ij_* ≡ {0,1} with *i* = 1, …, 3 (indicating rows) and *j* = 1, …, *L* (indicating column). The 0 value models homogeneity, while the 1 value models the presence of ITH at tumor position (*i*,*j*). Tumor budding (extendible to extramural venous invasion or perineural invasion) is represented by a different matrix (θ)*_ij_* ≡ {0,1}, where the 0 and 1 values represent, respectively, absence or presence of budding in position (*i*,*j*).

**Figure 2:**
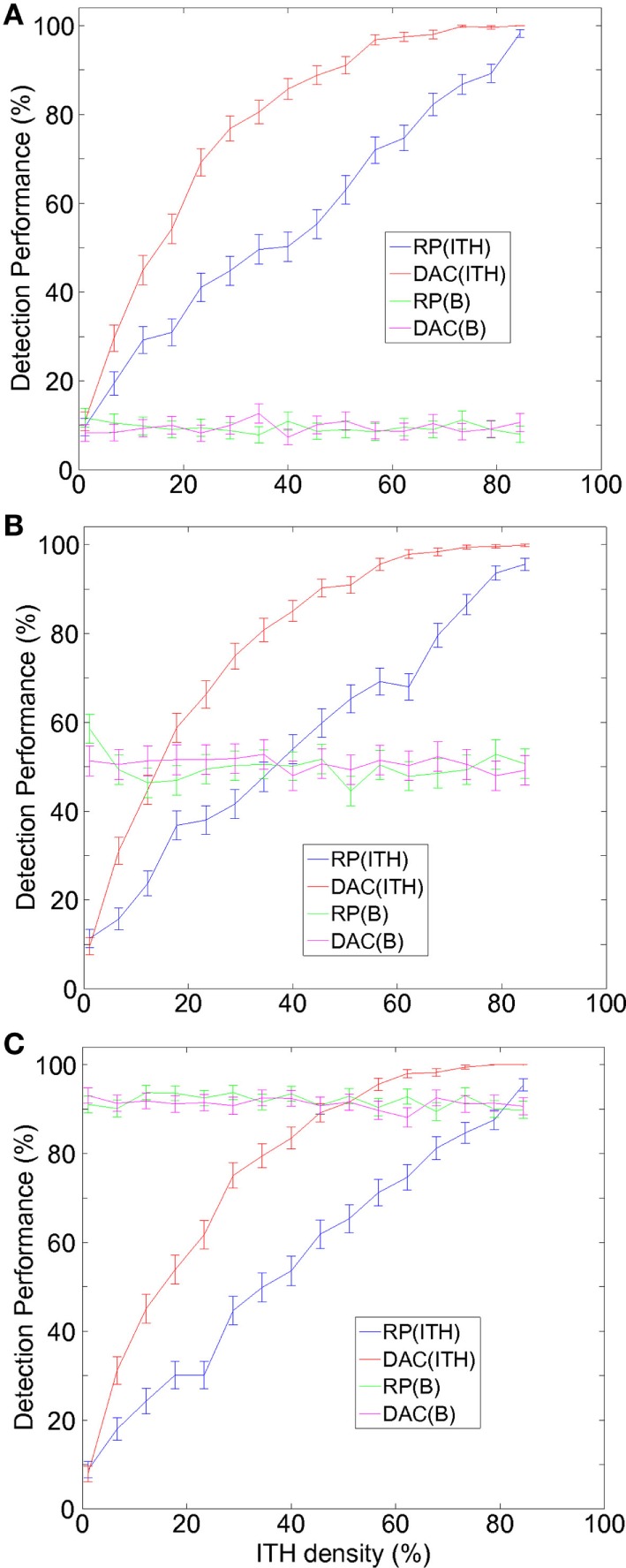
**A new divide-and-conquer (DAC) strategy outperforms RP in hollow viscera tumors, detecting better intratumor heterogeneity (ITH) and equally well budding**. **(A)** For low values of budding density, DAC detects more ITH than RP (red vs blue lines). DAC and RP performed equally well in detecting budding (magenta vs red lines). **(B)** Similar to panel **(A)**, but intermediate values of budding density. **(C)** Similar to panel **(A)**, but high values of budding density. **(A–C)** Percentage of either ITH or budding **(B)** detection (mean ± SE) as a function of the percentage of ITH density defining for each tumor. SE was calculated across *N* different repetitions of the same strategy and across *M* different tumors. Simulations parameters: *L* = 30 (side of 3 × *L* rectangle), *H* (number of sites with ITH) varying from 1 to 80 (or equivalently the ITH density ρ varying from approximately 0 to 89%), *B* (number of sites with budding) varying from 1 to 30 (or equivalently the budding density σ varying from approximately 0 to 100%), *N* = 50 (repetitions number for the two RP and DAC strategies), and *M* = 15 (number of simulated tumors). For the two strategies, RP and DAC, the total number of blocks for each repetition was equal to *Q*, which as explained in our previous approach, was modeling the laboratory costs. Hereon, we chose *Q* = 9 for both DAC and RP. For DAC, the *Q* sites consisted in three different parallel tissue stripes chosen at random (i.e., occupying *i* = 1, *i* = 2, and *i* = 3 sites and three random *j*’s, from *j* = 1, …, *L*). For RP, we first chose a site *J* randomly between 2 and *L* − 1, and after the sites (*i*,*J*), (*i*,*J* + 1), and (*i*,*J* − 1) with *i* = 1, 2, and 3.

Similar to our previous approach (10), two classes of ITH were modeled, random ITH (ranITH) and regional ITH (regITH). For both ranITH and regITH situations, ITH was simulated using an iterative method with *h* = 1, …, *H* steps and where the initial condition was for all the cases γ = 0. At each step *h*, a 2D position (*i*,*j*) is chosen at random and a value of (γ)_*ij*_ = 1 is assigned. After the *H* steps, the γ matrix is fixed and defines the tumor ITH configuration. The percentage of ITH density associated with a given tumor is defined as $\rho \equiv \frac{H}{3\times L}\times 100$ (c.f., *x*-axis in Figure 2).

For the ranITH situation, the matrix elements of γ were randomly generated at position (*i*,*j*), with no constraints for *i* and *j*, and this occurred for all the *H* steps. By contrast, for regITH, only at the first iteration (*h* = 1), the value of γ was assigned at position (*i*,*j*) with no constraints for *i* and *j* (using the same procedure as for ranITH), but for the following iterations (*h* ≥ 2), either the new chosen *i* or the new *j* was constrained to be necessarily a neighbor index of any of all the previously chosen *i* or *j*.

Budding was present only in tumor row of *i* = 1 (modeling the tumor invasion front). Budding was simulated using an iterative method with *b* = 1, …, *B* steps and where the initial condition was for all the cases θ = 0. At each step *b*, a random position *j* = 1, …, *L* was chosen and a value of (θ)_1_*_j_* = 1 was assigned. After the *B* steps, the θ matrix is fixed and defines the configuration of tumor budding. The percentage of budding density associated with a given tumor is defined as $\sigma \equiv \frac{B}{L}\times 100$.

For a given tumor, and after introducing fixed ITH and budding configurations defined by γ and θ, we repeated *N* times (and separately) two different strategies: routine protocol (RP, the one accepted in routine pathology) and our alternative, the DAC strategy. For each repetition and strategy, we calculated the number of successfully detected ITH and budding sites. Results were averaged across the *N* repetitions and also across *M* different tumors (each one with different ITH and budding configurations) (Figure 2).

## Take-Home Messages

Multisite tumor sampling is the best method for tumor sampling to detect ITH balancing high performance and sustainable cost. MSTS must be adapted to tumor shape and tumor location for an optimal performance.

## Author Contributions

JL and GP exposed the problem and provided an affordable solution; JC implemented the modeling approach; JL, GP, and JC wrote the final version of the manuscript and agreed with this submission.

## Conflict of Interest Statement

The authors declare that the research was conducted in the absence of any commercial or financial relationships that could be construed as a potential conflict of interest.

## References

[1] de la Fuente IM Elements of the cellular metabolic structure. Front Mol Biosci (2015) 2:16 10.3389/fmolb.2015.00016 25988183 PMC4428431

[2] Potter M Newport E Morten KJ. The Warburg effect: 80 years on. Biochem Soc Trans (2016) 44:1499–505. 10.1042/BST20160094 27911732 PMC5095922

[3] Alexandrov LB Nik-Zainal S Wedge DC Aparicio SA Behjati S Biankin AV Signatures of mutational processes in human cancer. Nature (2013) 500(7463):415–21. 10.1038/nature12477 23945592 PMC3776390

[4] Gerlinger M Rowan AJ Horswell S Larkin J Endesfelder D Gronroos E Intratumor heterogeneity and branched evolution revealed by multiregion sequencing. N Engl J Med (2012) 366:883–92. 10.1056/NEJMoa1113205 22397650 PMC4878653

[5] Alizadeh AA Aranda V Bardelli A Blanpain C Bock C Borowski C Toward understanding and exploiting tumor heterogeneity. Nat Med (2015) 21:846–53. 10.1038/nm.3915 26248267 PMC4785013

[6] Waclaw B Bozic I Pittman ME Hruban RH Vogelstein B Nowak MA. A spatial model predicts that dispersal and cell turnover limit intratumour heterogeneity. Nature (2015) 525(7568):261–4. 10.1038/nature14971 26308893 PMC4782800

[7] Hoefflin R Lahrmann B Warsow G Hübschmann D Spath C Walter B Spatial niche formation but not malignant progression is a driving force for intratumoural heterogeneity. Nat Commun (2016) 7:ncomms11845. 10.1038/ncomms11845 27291893 PMC4910022

[8] Mroz EA Rocco JW The challenges of tumor genetic diversity. Cancer (2016). 10.1002/cncr.30430 PMC5370554 27861749

[9] Rosai J Rosai and Ackerman’s Surgical Pathology. 10th ed Edinburgh: Mosby-Elsevier (2011).

[10] López JI Cortés JM A divide-and-conquer strategy in tumor sampling enhances detection of intratumor heterogeneity in pathology routine: a modeling approach in clear cell renal cell carcinoma. F1000Res (2016) 5:385 10.12688/f1000research.8196.2 27127618 PMC4830216

[11] Guarch R Cortés JM Lawrie CH López JI Multi-site tumour sampling (MSTS) significantly improves the performance of histological detection of intratumour heterogeneity in clear cell renal cell carcinoma (CCRCC). F1000Res (2016) 5:2020 10.12688/f1000research.9419.2 27635226 PMC5007747

[12] Cormen TH Leiserson CE Rivest RL Introduction to Algorithms. 2nd ed Cambridge: MIT Press (2001).

[13] Min D Yang W. A divide-and-conquer strategy to improve diffusion sampling in generalized ensemble simulations. J Chem Phys (2008) 128:094106. 10.1063/1.2834500 18331086

[14] Eisenstein M Cell sorting: divide and conquer. Nature (2006) 441(7097):1179–85. 10.1038/4411179a 16810261

[15] Kristensen VN Divide and conquer: the genetic basis of molecular subclassification of breast cancer. EMBO Mol Med (2011) 3:183–5. 10.1002/emmm.201100128 21394915 PMC3377072

[16] Fukumoto K Kikuchi E Mikami S Oqihara K Matsumoto K Miyajima A Tumor budding, a novel prognostic indicator for predicting stage progression in T1 bladder cancers. Cancer Sci (2016) 107:1338–44. 10.1111/cas.12990 27317460 PMC5021027

[17] Kobayashi K Matsumoto H Matsuyama H Fujii N Inoue R Yamamoto Y Clinical significance of CD44 variant 9 expression as a prognostic indicator in bladder cancer. Oncol Rep (2016) 36:2852–60. 10.3892/or.2016.5061 27599396

[18] Rogers AC Winter DC Heeney A Gibbons D Lugly A Puppa G Systematic review and meta-analysis of the impact of tumour budding in colorectal cancer. Br J Cancer (2016) 115:831–40. 10.1038/bjc.2016.274 27599041 PMC5046217

[19] Siegel RL Miller KD Jemal A. Cancer statistics, 2016. CA Cancer J Clin (2016) 66:7–30. 10.3322/caac.21332 26742998

[20] Gore JL Litwin MS Lai J Yano EM Madison R Setodji C Use of radical cystectomy for patients with invasive bladder cancer. J Natl Cancer Inst (2010) 102:802–11. 10.1093/jnci/djq121 20400716 PMC3245689

[21] Chandra A Griffiths D McWilliam LJ. Best practice: gross examination and sampling of surgical specimens from the urinary bladder. J Clin Pathol (2010) 63:475–9. 10.1136/jcp.2009.071191 20498023

[22] Amin MB Srigley JR Grignon DJ Reuter VE Humphrey PA Cohen MB Updated protocol for the examination of specimens from patients with carcinoma of the urinary bladder, ureter, and renal pelvis. Arch Pathol Lab Med (2003) 127:1263–79. 10.1043/1543-2165(2003)127<1263:UPFTEO>2.0.CO;2 14521469

[23] Lopez-Beltran A Bassi P Pavone-Macaluso M Montironi R. Handling and pathology reporting of specimens with carcinoma of the urinary bladder, ureter, and renal pelvis. Eur Urol (2004) 45:257–66. 10.1016/j.eururo.2003.09.018 15036668

[24] Eiró N Pidal I Fernandez-Garcia B Junquera S Lamelas ML del Casar JM Impact of CD68/(CD3+CD20) ratio at the invasive front of primary tumors on distant metastasis development in breast cancer. PLoS One (2012) 7:e52796. 10.1371/journal.pone.0052796 23300781 PMC3530508

[25] Thomsen MBH Nordentoft I Lamy P Hoyer S Vang S Hedegaard J Spatial and temporal clonal evolution during development of metastatic urothelial carcinoma. Mol Oncol (2016) 10:1450–60. 10.1016/j.molonc.2016.08.003 27582092 PMC5423216

[26] Angulo JC López JI Flores N Toledo JD. The value of tumour spread, grading and growth pattern as morphological predictive parameters in bladder carcinoma. A critical revision of the 1987 TNM classification. J Cancer Res Clin Oncol (1993) 119:578–93. 10.1007/BF01372721 8335677 PMC12200057

[27] Angulo JC López JI Ropero S. DNA methylation and urological cancer, a step towards personalized medicine: current and future prospects. Mol Diagn Ther (2016) 20:531–49. 10.1007/s40291-016-0231-2 27501813

[28] Amiri M Janssen F Kunst AE. The decline in stomach cancer mortality: exploration of future trends in seven European countries. Eur J Epidemiol (2011) 26:23–8. 10.1007/s10654-010-9522-9 21086022 PMC3018592

[29] Cancer Genome Atlas Research Network. Comprehensive molecular characterization of gastric adenocarcinoma. Nature (2014) 513(7517):202–9. 10.1038/nature13480 25079317 PMC4170219

[30] Hudler P. Challenges of deciphering gastric cancer heterogeneity. World J Gastroenterol (2015) 21:10510–27. 10.3748/wjg.v21.i37.10510 26457012 PMC4588074

[31] Carneiro F Oliveira C Suriano G Seruca R. Molecular pathology of familial gastric cancer, with an emphasis on hereditary diffuse gastric cancer. J Clin Pathol (2008) 61:25–30. 10.1136/jcp.2006.043679 17513507

[32] Oliveira C Pinheiro H Figueiredo J Seruca R Carneiro F. Familial gastric cancer: genetic susceptibility, pathology, and implications for management. Lancet Oncol (2015) 16:e60–70. 10.1016/S1470-2045(14)71016-2 25638682

[33] Lauwers GI Epithelial neoplasms of the stomach. In: Odze RD Goldblum JR, editors. Odze and Goldblum Surgical Pathology of the GI Tract, Liver, Biliary Tract, and Pancreas. Philadelphia: Elsevier Saunders (2015). p. 707–21.

[34] Robert ME Lamps L Lauwers GY Association of Directors of Anatomic and Surgical Pathology. Recommendations for the reporting of gastric carcinoma. Hum Pathol (2008) 39:9–14. 10.1016/j.humpath.2007.05.024 17967475

[35] Grigore AD Jolly MK Dongya D Farach-Carson MC Levine H. Tumor budding: the name is EMT. Partial EMT. J Clin Med (2016) 5:E51. 10.3390/jcm5050051 27136592 PMC4882480

[36] Koelzer VH Langer R Zlobec I Lugli A. Tumor budding in upper gastrointestinal carcinomas. Front Oncol (2014) 4:216. 10.3389/fonc.2014.00216 25177546 PMC4132482

[37] Jass JR O’Brien J Riddell RH Snover DC Association of Directors of Anatomic and Surgical Pathology Recommendations for the reporting of surgically resected specimens of colorectal carcinoma: association of directors of anatomic and surgical pathology. Am J Clin Pathol (2008) 129:13–23. 10.1309/6UHNC7MAD8KWNAWC 18089485

[38] Gibson KM Chan C Chapuis PH Dent OF Bokey L. Mural and extramural venous invasion and prognosis in colorectal cancer. Dis Colon Rectum (2014) 57:916–26. 10.1097/DCR.0000000000000162 25003286

[39] Mayo E Llanos AA Yi X Duan SZ Zhang L. Prognostic value of tumour deposit and perineural invasion status in colorectal cancer patients: a SEER-based population study. Histopathology (2016) 69:230–8. 10.1111/his.12936 26802566

[40] Dou R Nishihara R Cao Y Hamada T Mima K Masuda A MicroRNA let-7, T cells, and patient survival in colorectal cancer. Cancer Immunol Res (2016) 4:927–35. 10.1158/2326-6066.CIR-16-0112 27737877 PMC5115629

[41] Punt CJ Koopman M Vermeulen L. From tumour heterogeneity to advances in precision treatment of colorectal cancer. Nat Rev Clin Oncol (2016). 10.1038/nrclinonc.2016.171 27922044

[42] López JI Cortés JM Multi-site tumor sampling (MSTS): a new tumor selection method to enhance intratumor heterogeneity detection. Hum Pathol (2017). 10.1016/j.humpath.2017.02.010 28237785

[43] López JI Cortés JM A multi-site cutting device implements efficiently the divide and-conquer strategy in tumor sampling. F1000Res (2016) 5:1587 10.12688/f1000research.9091.2 27540472 PMC4965694

